# Zika virus infection during pregnancy: what, where, and why?

**DOI:** 10.3399/bjgp16X683917

**Published:** 2016-03

**Authors:** Rachael M Burke, Pranav Pandya, Eleni Nastouli, Philip Gothard

**Affiliations:** Core Medical Trainee, Hospital for Tropical Diseases, London.; University College London Hospitals, London.; Clinical Lead in Virology, University College London Hospitals, London.; Consultant Physician, Hospital for Tropical Diseases, London.

On 1 February 2016 the World Health Organization declared a Public Health Emergency of International Concern following reports of large clusters of microcephaly and Guillain-Barré Syndrome associated with an increase in cases of Zika virus (ZIKV) infection in French Polynesia (2014) and Brazil (2015– 2016).^1^ The Committee emphasised that there was *‘... no public health justification for restrictions on travel or trade’* and the main interventions were to control mosquito populations and prevent bites in pregnant women. Why has this happened and how might it affect patients attending primary care in the UK?

ZIKV was first isolated from a Rhesus monkey in Uganda in 1947.^2^ The following year it was identified in Aedes mosquitoes, which differ from malaria-transmitting Anopheles mosquitoes by biting during the day. ZIKV has been found throughout Africa and South East Asia where infection is asymptomatic or produces a mild febrile illness and rash which goes undiagnosed. The first outbreak was not recorded until 2007 when three-quarters of the population of Yap Island in Micronesia became infected.^3^

The current epidemic of ZIKV infection began in early 2015 in northeastern Brazil. Since then ZIKV transmission has been confirmed in 35 countries.^4^ One theory is that ZIKV was carried to Brazil by infected Pacific Islanders visiting an international canoeing event in Rio de Janeiro in August 2014. In September 2015 clinicians working in Pernambuco state noticed an increase in newborn babies with microcephaly. The Ministry of Health quickly established a register and within 3 months recorded 4180 suspected cases, including 68 deaths, compared to a total of 147 reports in the whole of 2014.^5^ A review of the first 35 cases noted that 74% of mothers reported a rash during pregnancy and 71% of infants had severe microcephaly.^6^ ZIKV RNA was detected in the amniotic fluid of two mothers and from the brain of a baby who died shortly after birth.^7^ Taken together these data indicate a strong association between ZIKV infection during pregnancy and microcephaly, although a causal relationship is yet to be proven.

ZIKV infection has an incubation period of 3–12 days. Patients may present with a fever, rash, arthralgia, and conjunctivitis. The illness is self-limiting and lasts for up to a week. Severe cases are uncommon. It can be difficult to distinguish ZIKV infection from other viral illnesses such as dengue and chikungunya, which are also transmitted by Aedes mosquitoes. Population seroprevalence studies from the outbreak in Micronesia showed that 80% of ZIKV infections were asymptomatic,^8^ which presents a diagnostic problem in pregnancy if ZIKV crosses the placenta.

## PRACTICAL ADVICE

**Figure 1.**
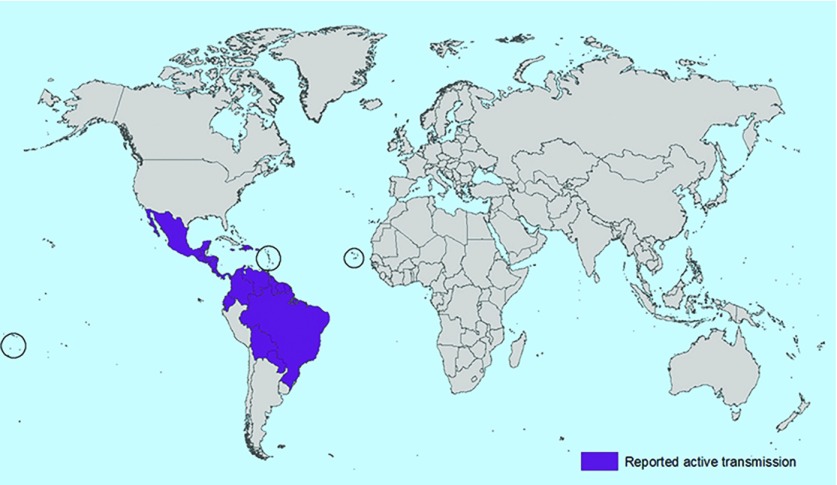
***Centers for Disease Control and Prevention map of the geographic distribution of Zika virus as of 3 February 2016. Source: http://www.cdc.gov/zika/images/zik-world-map_active_02-03-2016_web.jpg.***

Public Health England (PHE) and the Royal College of Obstetrics and Gynaecology released joint interim guidelines on 29 January 2016^9^ followed by PHE and the Royal College of General Practitioners with primary care guidelines on 4 February 2016.^10^ They recommend that pregnant women who present with symptoms consistent with ZIKV infection within 2 weeks of travel to an area of active transmission (Figure 1) should be tested for ZIKV.

There is a wide differential diagnosis of illnesses in returning travellers, including malaria, and patients with symptoms should be discussed with a physician experienced in their assessment. EDTA plasma and urine samples from symptomatic patients should be sent to the Rare and Imported Pathogens Laboratory at Porton Down (Box 1). Testing involves reverse transcription polymerase chain reaction (RT-PCR) of blood and urine. Antibody testing is not yet available in the UK and is currently less reliable due to potential cross-reaction with similar viruses, such as dengue and yellow fever. This may change soon and for now PHE recommend that all patients suspected of ZIKV infection, and any pregnant women exposed to ZIKV, should have a serum sample collected and saved. While these interim guidelines are welcome, it is important to be aware that there are limited data available to make conclusive recommendations. Pre-test counselling is important, particularly as the full characteristics of the tests are unknown and false-negative results are possible.

Box 1. **Further information**

**Clinical assessment of returning travellers with symptoms**

- Hospital for Tropical Diseases, Mortimer Market Centre, Capper Street, London WC1E 6JB
  Walk-in clinic Monday to Friday 9 am to 4 pm. On-call tropical registrar (24 hours) via UCLH switchboard 020 3456 7890; http://www.thehtd.org

**Pre-travel telephone advice to health professionals**

- National Travel Health Network and Centre (NaTHNaC): 0845 602 6712
  http://www.uclh.nhs.uk/ourservices/servicea-z/htd/nathnac/pages/home.aspx

**Practical guidelines for managing Zika virus exposure in pregnancy**

- Royal College of Obstetricians and Gynaecologists
  https://www.rcog.org.uk/en/news/interim-clinical-guidelines-on-zika-virus-infection-and-pregnancy/
- Royal College of General Practitioners
  https://www.gov.uk/government/uploads/system/uploads/attachment_data/file/497767/Zika_virus_guidance_for_primary_care_February_2016_FINAL.pdf

**How to send samples for Zika virus testing**

- Rare and Imported Pathogens Laboratory
  https://www.gov.uk/government/collections/rare-and-imported-pathogens-laboratory-ripl

**The risk of Zika virus and other infections in pregnancy**

- Public Health England
  https://www.gov.uk/guidance/zika-virus

**General information**

- European Centre for Disease Prevention and Control
  http://ecdc.europa.eu/en/healthtopics/zika_virus_infection/Pages/index.aspx
- US Center for Disease Control and Prevention
  http://www.cdc.gov/zika/

All pregnant women who have potentially been exposed to ZIKV should be referred to their local maternity unit for 4-weekly fetal ultrasound scan (USS) examinations. This includes asymptomatic patients, those who have had symptoms outside the testing window and patients who have tested negative for ZIKV by RT-PCR. Microcephaly is a head circumference below the 2.5th centile for gestational age and standard fetal USS is a sensitive screening test for this and other intracranial abnormalities such as ventriculomegaly and calcification.

Pregnant women with a positive ZIKV RT-PCR or with concerning findings on USS should be referred to a fetal medicine service for evaluation and follow up. This may involve a detailed USS and possibly amniocentesis from 15 weeks to test for ZIKV and other causes of neonatal infection in the amniotic fluid. Fetal brain MRI may detect abnormalities not seen on USS. It is important to remember that microcephaly and other intracranial anomalies may be caused by a number of disorders unrelated to ZIKV. PHE has clear guidelines for evaluating pregnant women with a rash, in particular, advice on when to test for rubella, varicella, or parvovirus B19.^11^

Pregnant women should consider avoiding travel to areas with ongoing ZIKV outbreaks and seek advice from a travel health specialist. The first trimester probably carries the greatest risk of microcephaly. If travel is unavoidable they should be advised to take great care to protect against daytime mosquito bites by covering up and using insect repellents.

There is emerging evidence of ZIKV transmission through sexual intercourse.^12^ It is not known how long ZIKV persists in semen and UK and US guidance differs, however this is unlikely to be a common route of transmission. For simplicity our advice is to follow the US Centers for Disease Control and Prevention guidance to abstain from sex or use condoms for the duration of the pregnancy if a male partner has been in a Zika virus area.^9^

ZIKV poses significant challenges in the counselling process in pregnancy as limited evidence exists to about the proportion of infected patients who are asymptomatic and have brief, low level viraemia, the risk to the fetus relative to the time of infection, the reliability and significance of laboratory tests, and the likelihood of the child developing neurological sequalae. These questions are the subject of ongoing research, but in the meantime, 1.4 million UK travellers visit countries with ongoing ZIKV transmission each year and of these we estimate that around 280 000 are women of child-bearing age. If only a small number of these are pregnant that represents several thousand women who may consider themselves at risk. Fortunately the limited data suggest that the risks to the baby are very low. However pregnancy is often an anxious time and GPs can help expectant mothers by being aware of the latest guidelines and where to seek help.
